# Predictive role of insulin resistance surrogates in gallstone disease

**DOI:** 10.1097/MD.0000000000041478

**Published:** 2025-04-04

**Authors:** Jia Liu, Zixuan Hu, Dele Bo, Xiaohui Zhang, Zuoyang Zhang, Xueqing Liu

**Affiliations:** a Physical Examination Center, The Second Hospital of Hebei Medical University, Shijiazhuang, Hebei, China; b Department of Hepatobiliary Surgery, The Second Hospital of Hebei Medical University, Shijiazhuang, Hebei, China.

**Keywords:** density lipoprotein cholesterol ratio, gallstone disease, glucose, insulin resistance, triglyceride, triglycerides/high, TyG related to BMI, TyG related to WC

## Abstract

Several studies have confirmed the association between insulin resistance (IR) surrogates and the risk of cardiovascular disease. This study aimed to investigate the correlation between 4 IR surrogates: triglyceride-glucose index (TyG), triglyceride-glucose index with waist circumference (TyG-WC), triglyceride-glucose index with body mass index (TyG-BMI), and triglycerides/high-density lipoprotein cholesterol ratio, and the risk of gallstone disease. This retrospective cross-sectional study involved a total of 19,696 participants who were divided into gallstone and non-gallstone groups based on ultrasound findings. Biochemical parameters and ultrasound results were collected and statistically analyzed. Nonparametric *U* test, Chi-square test, and subgroup analysis were used to examine the differences in expression of the 4 IR surrogates between the 2 groups. Logistic regression models and receiver operating characteristic curves were employed to evaluate the relationships and predictive ability of the 4 surrogates for gallstone disease. The levels of the 4 surrogates of IR were significantly higher in individuals with gallstone disease (GSD) compared to those without GSD (*P* < .001). After adjusting for age, gender, and personal medical history, TyG-WC and TyG-BMI emerged as significant predictors of gallstones in both genders. The predictive ability of these IR surrogates was stronger for gallstone disease in females than in males. In females, the area under the curve for TyG-WC and TyG-BMI were 0.683 and 0.629, respectively, while for males, these values were 0.544 and 0.528. When age was included with TyG-WC and TyG-BMI to predict GSD, the area under the curve values increased to 0.734 and 0.733 for females, and 0.684 and 0.682 for males. The study found that TyG-WC and TyG-BMI were identified as independent risk factors for the prevalence of GSD. Additionally, combined with age, TyG-WC and TyG-BMI showed good predictive value for the prevalence of GSD in females.

## 1. Introduction

Gallstone disease is a common gastrointestinal disorder worldwide, causing bile duct pain and potential complications like cholecystitis, pancreatitis, and cholangitis. In Western countries, approximately 10% to 15% of adults are estimated to have gallstones, with over 20 million affected individuals in the United States.^[1]^ A population-based screening study in China in 2012 revealed gallstone disease rates of 3.8% in South China and 6.1% in North China.^[2]^ While various studies have explored risk factors for gallbladder stones, conclusive evidence remains elusive. Multiple factors have been linked with an elevated risk of gallstones, indicating that age, gender, family history, and pregnancies are crucial in the formation of gallstones.^[3]^ Continuous emergence of new factors contributes to the identification of risks. Insulin resistance (IR) is a prominent risk factor, particularly in relation to obesity, diabetes, and metabolic syndrome, which are recognized as independent elements that increase the risk of developing stones. IR is viewed as a fundamental pathophysiology underlying stone formation in these individuals.^[4]^ Conventional methods for assessing IR, such as the hyperinsulinemic-euglycemic clamp and the homeostasis model assessment of IR (HOMA-IR) index, are not commonly utilized in primary healthcare settings due to their invasive, complex, and impractical.^[5]^

Early diagnosis and effective screening of high-risk populations for GSD are crucial imperatives. Recent studies have highlighted the significant association between obesity and IR, particularly focusing on the product of fasting TG and glucose levels (TyG) and TyG-related parameters.^[6,7]^ These markers can be measured using standard biochemical tests as a simple and reliable indicator for assessing IR. Numerous clinical and epidemiological studies have demonstrated a link between IR surrogates and conditions such as prostate cancer and kidney stones.^[8,9]^ However, there is a gap in existing research regarding the comparative predictive capabilities of the TyG index and other related factors for gallstones. Therefore, our study aims to explore the relationship between the TyG index and gallstones, while also comparing TyG with other parameters (triglyceride-glucose index with waist circumference [TyG-WC] and triglyceride-glucose index with body mass index [TyG-BMI]) as well as triglycerides/high-density lipoprotein cholesterol ratio (TG/HDL-c) in relation to gallstone occurrence among individuals undergoing physical examinations in Hebei province, China.

## 2. Materials and methods

**Figure 1.**
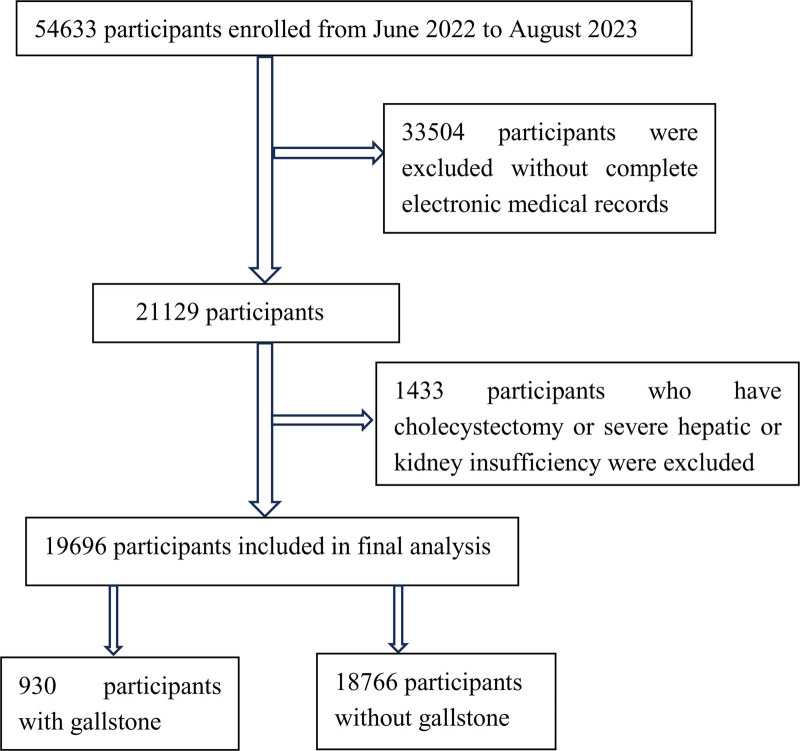
Flow chart of the study population for the gallstone group.

This retrospective, observational study was conducted at a single center, involving participants from the physical examination center of the Second Hospital of Hebei Medical University from June 2022 to July 2023. The study sample comprised asymptomatic individuals who underwent routine physical examinations, excluding those with symptomatic conditions. Patients with missing data on biochemical indicators, previous cholecystectomy, or severe hepatic or kidney insufficiency were excluded from the analysis. Figure 1 depicts the flowchart of the participants included in this study.

The study recorded various clinical characteristics of all subjects, such as age, gender, body mass index (BMI), waist circumference (WC), smoking status, drinking history, blood pressure, fasting blood glucose, serum total cholesterol (TC), triglyceride (TG), low-density lipoprotein cholesterol, HDL-c, uric acid (UA), and abdominal ultrasound. The individuals were identified using ultrasound examination as individuals with gallstones, while the control group was comprised of lacking. Peripheral blood samples are usually obtained by venipuncture from individuals who have fasted the night before and the laboratory tests were performed within 2 hours of collection. Type 2 diabetes was defined as self-reported physician-diagnosed diabetes mellitus. Hypertension was defined as having a systolic blood pressure of 140 mm Hg or higher and/or a diastolic blood pressure of 90 mm Hg or higher, or being on antihypertensive medication. The diagnosis of fatty liver was determined based on findings from abdominal ultrasound. All subjects were diagnosed with gallbladder stone by abdominal ultrasonography examination for 2 experienced doctors. Gallstones are identified by the presence of strong echoes within the gallbladder lumen or gallbladder sludge accompanied by acoustic shadowing and signs of gallstones.^[10]^ This study was approved by the Ethics Committee of the Second Hospital of Hebei Medical University. The TyG index was determined using the formula: Ln[TG (mg/dL)·fasting glucose (mg/dL)/2]. The TyG-WC value was obtained by multiplying the TyG index by the WC. The TG to HDL-c ratio (TG/HDL-c).

### 2.1. Statistical analysis

Statistical analyses were performed using SPSS 27.0 (SPSS Inc., Chicago, IL). The study utilized median (interquartile range) to represent the general measurement data of the research subjects. A nonparametric *U* test was employed for comparing the 2 groups, while count data was presented as n (%) and compared using the χ^2^ test. Spearman correlation analyses were conducted to assess the 4 IR surrogates and the gallstone traditional risk factors. Multivariable logistic regression models were utilized to assess the correlation between TyG, TyG-WC, TyG-BMI, and TG/HDL-c with GSD. The 4 parameters were examined as continuous variables. Three different models were created to control for possible confounders. Model 1 was unadjusted, while model 2 accounted for age, gender, smoking, and alcohol consumption, and model 3 adjusted for age, gender, smoking, drinking, and components of hypertension, diabetes mellitus type 2 (T2DM), hyperlipidemia, fatty liver. Receiver operating characteristic (ROC) curves were utilized to evaluate predictive validity and determine the ideal cutoff values for the 4 IR surrogates in identifying gallstones in the study participants. The ROC curve for each surrogate in predicting gallstones was analyzed for all individuals. Additionally, separate ROC curves were generated for males and females to evaluate the efficacy of surrogate in identifying gallstones within subgroups. Significant pairwise comparisons were conducted for the area under the curve (AUC) of the 4 parameters. A two-tailed *P*-value < .05 was deemed significant in all analyses.

## 3. Results

### 3.1. Basic characteristics and laboratory data of the study population

Table 1 presents the baseline characteristics of the complete population, categorized by the occurrence of gallstone. Subjects were divided according to the results of abdominal ultrasonography into the gallstones group (n = 930) and the non-gallstones group (n = 18766). The gallstone group showed a higher percentage of males, individuals who smoke, as well as an increased incidence of hypertension, T2DM, and fatty liver. Additionally, they exhibited raised levels of age, BMI, WC, FPG, TG, HDL-C, UA, TyG, TyG-BMI, TyG-WC, and TG/HDL-C at baseline in contrast to those who did not have gallstones.

**Table 1:** Basic characteristics and laboratory data of the study population.

Variables	All (n = 19,696)	Gallstone (n = 930)	Non-gallstone (n = 18766)	*P*
Age, median (IQR), years	*48*	59	47	<.001
Sex (men, n%)	9151 (46.5%)	552 (59.4%)	8599 (45.8%)	<.001
BMI (kg/m^2^)	24.35	25.36	24.30	<.001
WC (cm)	86	91	86	<.001
Smoking (n%)	2969 (15.1%)	166 (17.8%)	2803 (14.9%)	.016
Drinking (n%)	5447 (27.7%)	257 (27.6%)	5190 (27.7%)	.998
Hypertension (n%)	*3284 (16.7%*)	320 (34.4%)	2964 (15.8%)	<.001
T2DM (n%)	1337 (6.8%)	153 (16.5%)	1184 (6.3%)	<.001
Hyperlipidemia (n%)	165 (0.8%)	6 (0.6%)	159 (0.8%)	.710
Fatty liver (n%)	5694 (28.9%)	340 (36.6%)	5354 (28.5%)	<.001
FPG, median (IQR), mmol/L	4.98	5.22	4.97	<.001
TG, median (IQR), mmol/L	1.19	1.33	1.19	<.001
TC, median (IQR), mmol/L	4.74	4.74	4.74	.475
LDL-C, median (IQR), mmol/L	2.73	2.75	2.73	.906
HDL-C, median (IQR), mmol/L	1.38	1.29	1.39	<.001
UA, median (IQR), mmol/L	319	335	318	<.001
TyG, median (IQR)	8.49	8.65	8.49	<.001
TyG-WC, median (IQR)	736.02	795.41	732.90	<.001
TyG-BMI, median (IQR)	207.97	222.95	207.31	<.001
TG/HDL-C, median (IQR)	1.97	2.37	1.95	<.001

BMI = body mass index, FPG = fasting plasma glucose, HDL-C = high-density lipoprotein cholesterol, LDL-C = low-density lipoprotein cholesterol, TC = total cholesterol, T2DM = diabetes mellitus type 2, TG = triglyceride, TYG = triglyceride glucose, TyG-BMI = TyG related to BMI, TG/HDL-C = triglyceride-to-HDL cholesterol ratio, TyG-WC = TyG related to WC, UA = uric acid, WC = waist circumference.

### 3.2. Spearman correlation analysis between the 4 IR surrogates and the gallstone traditional risk factors

**Figure 2.**
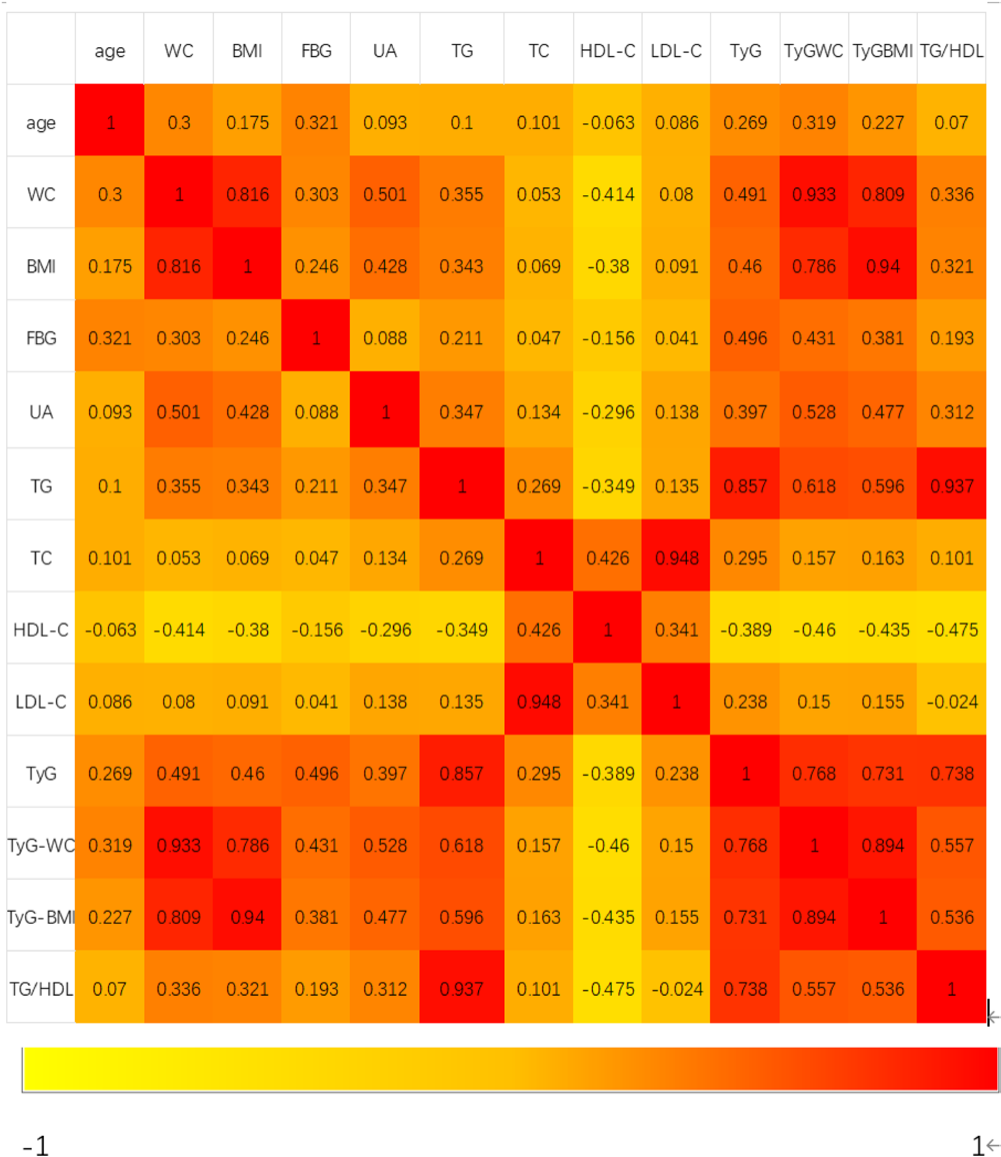
Spearman correlations between the 4 IR surrogates and gallstone traditional risk clinical parameters. IR = insulin resistance.

Correlation between the 4 IR surrogates and the gallstone traditional risk factors was examined using Spearman correlation analysis (Fig. 2). The results indicated a positive correlation between the 4 surrogates for variables such as age, WC, BMI, UA, and TC. Conversely, a negative correlation was observed between the TyG index and HDL-c.

### 3.3. Subgroup analysis of the 4 IR surrogates in non-gallstone and gallstone groups

Subgroup analysis was conducted for the 4 surrogate markers of IR in both non-gallstone and gallstone groups, stratified by gender, age, and BMI as outlined in Table 2. Across both genders, TyG-WC and TyG-BMI displayed notably higher values in the gallstone cohort compared to the non-gallstone cohort, while TyG and TG/HDL-c levels were elevated exclusively in females with gallstones (*P* < .05). Levels of TyG, TyG-WC, TyG-BMI, and TG/HDL-C were significantly higher in individuals with gallstones regardless of age, in comparison to those without gallstones. Furthermore, individuals with gallstones exhibited higher levels of these markers at normal and overweight BMI categories compared to those without gallstones (*P* < .001), with no significant difference observed at the obese level.

**Table 2:** Differences in subgroup analysis of 4 IR surrogate indicators between the non-GSD group and the GSD group.

	Non-GSD	GSD			Non-GSD		GSD		
Gender	Male			*P*-value	Female			*P*-value	
TyG	8.70 (8.33–9.10)	8.73 (8.39–9.11)		.122	8.30 (7.96–8.68)		8.56 (8.27–8.94)	<.001	
TyG-WC	817.81 (749.69–888.38)	833.11 (766.69–910.56)		<.001	657.19 (596.56–730.99)		725.72 (659.58–806.20)	<.001	
TyG-BMI	224.87 (203.23–249.02)	229.06 (205.83–252.75)		.026	190.55 (169.76–215.39)		213.01 (189.77–237.63)	<.001	
TG/HDL-C	2.57 (1.71–3.93)	2.67 (1.79–3.86)		.552	1.56 (1.10–2.34)		2.01 (1.40–2.99)	<.001	

Age	≤39 year			40–59 year			≥60 year		
	Non-GSD	GSD	*P*-value	Non-GSD	GSD	*P*-value	Non-GSD	GSD	*P*-value
TyG	8.20 (7.88–8.61)	8.43 (7.00–8.94)	.005	8.57 (8.20–8.97)	8.63 (8.34–9.03)	.002	8.67 (8.33–9.04)	8.73 (8.39–9.10)	.044
TyG-WC	656.23 (585.59–764.09)	710.80 (644.30–833.69)	<.001	744.22 (658.65–836.53)	779.58 (688.63–857.73)	<.001	790.29 (718.52–860.57)	810.98 (743.42–884.34)	<.001
TyG-BMI	187.80 (166.44–218.39)	210.86 (185.42–244.35)	<.001	211.75 (188.12–238.68)	219.98 (198.09–247.92)	<.001	218.34 (196.14–242.48)	226.46 (203.65–249.79)	<.001
TG/HDL-C	1.54 (1.05–2.50)	2.01 (1.28–3.72)	.001	2.12 (1.41–3.36)	2.44 (1.63–3.55)	<.001	2.23 (1.55–3.28)	2.38 (1.67–3.41)	.015

BMI	<24 kg/m^2^			24–28 kg/m^2^			≥28 kg/m^2^		
	Non-GSD	GSD	*P*-value	Non-GSD	GSD	*P*-value	Non-GSD	GSD	*P*-value
TyG index	8.22 (7.92–8.58)	8.41 (8.09–8.71)	<.001	8.65 (8.30–9.01)	8.75 (8.42–9.13)	<.001	8.87 (8.53–9.27)	8.87 (8.56–9.24)	.960
TyG-WC	641.77 (585.96–705.71)	689.42 (638.53–747.67)	<.001	784.45 (721.82–843.96)	806.23 (757.84–856.43)	<.001	895.03 (831.35–960.86)	909.44 (852.85–962.82)	.121
TyG-BMI	179.20 (164.11–193.17)	188.02 (175.22–199.06)	<.001	222.83 (210.56–236.13)	227.43 (214.85–238.84)	<.001	226.72 (252.10–285.01)	265.45 (252.84–280.17)	.801
TG/HDL-C	1.45 (1.04–2.11)	1.68 (1.26–2.42)	<.001	2.39 (1.65–3.57)	2.65 (1.81–3.78)	<.001	3.08 (2.08–4.44)	2.85 (2.10–4.21)	.237

GSD = gallstone disease, IR = insulin resistance, TyG = triglyceride glucose, TyG-BMI = TyG related to BMI, TG/HDL-C = triglyceride-to-HDL cholesterol ration, TyG-WC = TyG related to WC, WC = waist circumference.

### 3.4. Logistic regression models of the 4 IR surrogates in relation to the risk of gallstones in different models

A univariate logistic regression analysis revealed associations between the 4 IR surrogate with gallstones. The 4 IR surrogates remained significant even after accounting for various gallstone risk factors such as age, gender, smoking, drinking, and personal disease history. Prior to adjustment, TyG exhibited the highest odds ratio (OR) among the 4 IR surrogates (1.641, 95% CI 1.481–1.817). Following adjustments, TyG continued to demonstrate the highest OR among the 4 IR surrogates (1.020, 95% CI 1.051–1.375) (Table 3).

**Table 3:** Multivariable analysis of the association between the 4 IR surrogates and GSD.

	Model 1		Model 2		Model 3	
	OR (95% CI)	*P*-value	OR (95% CI)	*P*-value	OR (95% CI)	*P*-value
TyG	1.641 (1.481–1.817)	<.001	1.327 (1.181–1.490)	<.001	1.020 (1.051–1.375)	.007
TyG-WC	1.003 (1.003–1.004)	<.001	1.002 (1.002–1.003)	<.001	1.002 (1.001–1.003)	<.001
TyG-BMI	1.008 (1.007–1.010)	<.001	1.006 (1.004–1.008)	<.001	1.005 (1.003–1.007)	<.001
TG/HDL	1.088 (1.046–1.132)	<.001	1.076 (1.028–1.126)	.002	1.050 (0.997–1.106)	.066

GSD = gallstone disease, IR = insulin resistance, T2DM = diabetes mellitus type 2, TyG = triglyceride glucose, TyG-BMI = TyG related to BMI, TG/HDL-C = triglyceride-to-HDL cholesterol ration, TyG-WC = TyG related to WC, WC = waist circumference.

Model 1: non-adjusted model.

Model 2: adjusted for age, gender, smoking, drinking.

Model 3: adjusted for age, gender, smoking, drinking, hypertension, T2DM, hyperlipidemia, and fatty liver.

### 3.5. Subgroup analysis stratified by sex revealed differences

To determine the consistency of the relationship between the 4 IR surrogates and risk of gallstones, we conducted stratified analyses (Table 4). In the non-adjusted model, TyG-WC was significantly predicted gallstones in both genders. TyG was most strongly associated with gallstones, the OR for GSD was 2.269 in females (*P* < .001). In Model 2, after adjusting for age, smoking, and drinking, we found TyG was the most strongly associated with gallstones, the OR for gallstones was 1.272 in males (*P* = .002) and 1.368 (*P* = .002) in females. The TyG-WC and TyG-BMI were significantly predicted gallstones in both genders. The TG/HDL-C was significant only in the female group. After adjusting for age, gender, smoking, drinking, hypertension, T2DM, hyperlipidemia, and fatty liver only TyG-WC and TyG-BMI significantly predicted gallstones in both genders.

**Table 4:** Subgroup analysis by gender between the 4 IR surrogates and GSD.

		Model 1		Model 2		Model 3	
		OR (95% CI)	*P*-value	OR (95% CI)	*P*-value	OR (95% CI)	*P*-value
TyG	Males	1.127 (0.976–1.302)	.103	1.272 (1.094–1.478)	.002	1.186 (1.000–1.408)	.051
	Females	2.269 (1.920–2.681)	<.001	1.368 (1.122–1.668)	.002	1.181 (0.941–1.482)	.151
TyG-WC	Males	1.001 (1.001–1.002)	<.001	1.002 (1.001–1.003)	<.001	1.002 (1.001–1.003)	<.001
	Females	1.005 (1.004–1.006)	<.001	1.003 (1.002–1.004)	<.001	1.002 (1.001–1.003)	<.001
TyG-BMI	Males	1.002 (1.000–1.005)	.059	1.005 (1.003–1.008)	<.001	1.005 (1.002–1.008)	<.001
	Females	1.013 (1.010–1.015)	<.001	1.007 (1.004–1.010)	<.001	1.005 (1.001–1.008)	.005
TG/HDL	Males	0.988 (0.926–1.054)	.708	1.055 (0.995–1.119)	.074	1.042 (0.978–1.111)	.200
	Females	1.193 (1.102–1.291)	<.001	1.054 (1.020–1.196)	<.001	1.061 (0.969–1.161)	.203

GSD = gallstone disease, IR = insulin resistance, TyG = triglyceride glucose; TyG-BMI = TyG related to BMI, TG/HDL-C = triglyceride-to-HDL cholesterol ration, TyG-WC = TyG related to WC, WC = waist circumference.

Model 1: non-adjusted model.

Model 2: adjusted for age, gender, smoking, drinking.

Model 3: adjusted for age, gender, smoking, drinking, hypertension, T2DM, hyperlipidemia, and fatty liver.

### 3.6. Predictive value of the 4 IR surrogates for gallstone

Table 5 presents the AUC results for various indicators related to the 4 IR surrogates of gallstones. Among the 4 IR surrogates, the TyG-WC demonstrated the highest predictive ability with an AUC value of 0.631 (95% CI 0.615–0.648). Following closely behind were TyG-BMI with an AUC of 0.611 (95% CI 0.594–0.628), TyG with an AUC of 0.595 (95% CI 0.577–0.612) and TG/HDL-C with an AUC of 0.585 (95% CI 0.568–0.603). The optimal cutoff points for predicting gallstone were determined to be 8.37 for TyG, 212.09 for TyG-BMI, 709.89 for TyG-WC, and 1.71 for TG/HDL-C.

**Table 5:** Performance of the 4 surrogates in gallstone of ROC curve analysis.

	AUC	95% CI	*P*-value	Sensitivity	Specificity	Youden index	Cutoff value
TyG	0.595	0.577–0.612	<.001	73.5	57.7	0.159	8.37
TyG-WC	0.631	0.615–0648	<.001	76.3	56.0	0.203	709.89
TyG-BMI	0.611	0.594–0.628	<.001	62.5	44.9	0.176	212.09
TG/HDL-C	0.586	0.568–0.603	<.001	71.8	58.4	0.134	1.71

BMI = body mass index; 95% CI = 95% confidence interval, FPG = fasting plasma glucose, ROC = receiver operating characteristic, TG = triglyceride, TyG = triglyceride glucose, TyG-BMI = TyG related to BMI, TG/HDL-C = triglyceride-to-HDL cholesterol ration, TyG-WC = TyG related to WC, WC = waist circumference.

**Figure 3.**
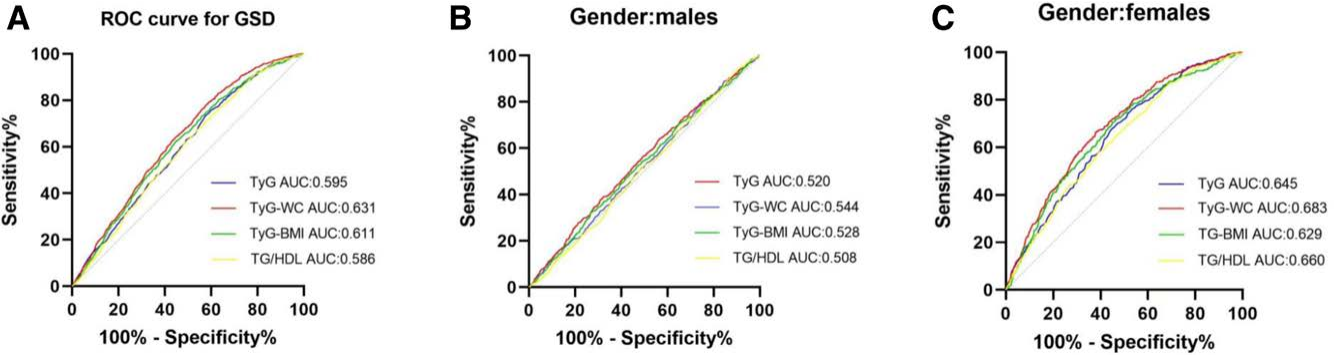
Receiver operating characteristic (ROC) curve analysis of TyG, TyG-WC, TyG-BMI, and TG/HDL-c in all participants (A), in males (B), and females (C). TyG = triglyceride-glucose index, TyG-BMI = triglyceride-glucose index with body mass index, TG/HDL-c = triglycerides/high-density lipoprotein cholesterol ratio, TyG-WC = triglyceride-glucose index with waist circumference.

As shown in Figure 3, the 4 surrogate markers for IR demonstrated better predictive ability for gallstone disease in women compared to men. TyG-WC exhibited the most reliable predictive power, with TG/HDL-C showing a slightly stronger predictive capability than TyG alone. The AUC for TyG-WC was 0.683. Despite attempts to combine TyG-WC and TG/HDL-C to forecast gallstone disease, there was no enhancement in predictive performance. However, incorporating age into the TyG, TyG-WC, TyG-BMI, and TG/HDL-C combination elevated the predictive accuracy to 72.7%, 73.4%, 73.3%, and 72.5%, respectively for gallstone disease prevalence in females. The predictive accuracies of the 4 IR surrogates in predicting gallstone prevalence were superior to those in males (Table 6).

**Table 6:** Performance of the 4 surrogates in gallstone of ROC curve analysis by sex.

							
Females	AUC	95% CI	*P*-value	Males	AUC	95% CI	*P*-value
TyG + Age	0.727	0.702–0.751	<.001	TyG + Age	0.680	0.658–0.702	<.001
TyG-WC + Age	0.734	0.710–0.758	<.001	TyG-WC + Age	0.684	0.662–0.706	<.001
TyG-BMI + Age	0.733	0.709–0.757	<.001	TyG-BMI + Age	0.682	0.661–0.704	<.001
TG/HDL-C + Age	0.725	0.700–0.758	<.001	TG/HDL-C + Age	0.679	0.657–0.701	<.001

AUC = area under the curve, BMI = body mass index, 95% CI = 95% confidence interval, FPG = fasting plasma glucose, HDL-c = high-density lipoprotein cholesterol, ROC = receiver operating characteristic, TG = triglyceride, TyG = triglyceride glucose, TyG-BMI = TyG related to BMI, TG/HDL-C = triglyceride-to-HDL cholesterol ration, TyG-WC = TyG related to WC, WC = waist circumference.

## 4. Discussion

This study conducted a cross-sectional analysis to thoroughly examine the correlations between the 4 IR surrogates and the risk of gallstone disease, considering age, sex, and weight status. Our findings suggest that various surrogates may have varying predictive capabilities for gallbladder stones. TyG index, TyG-WC, TyG-BMI, and TG/HDL-C were significantly increased in the gallstone group compared to the non-gallstone group. This association remained consistent across different age groups. TyG-WC and TyG-BMI were significant in both males and females, while the TyG and TG/HDL-C were significant only in the female group. The 4 IR surrogates were significant in non-obese people. Additionally, TyG-WC and TyG-BMI showed better predictive value for the prevalence of gallstones in females compared to males.

The diagnosis of GSD is simple, but it often presents with no clinical symptoms and is frequently overlooked by individuals. Numerous studies have demonstrated that IR and hyperinsulinemia are common in cholesterol stones which can contribute to the development or exacerbation of gallbladder stones. Insulin promotes gallstone formation by increasing hydroxy-3-methylglutaryl-CoA reductase activity and stimulating bile acid-independent flow of bile to the liver.^[11]^ Central to this process are visceral obesity and hepatic IR, which may promote cholesterol bile supersaturation and facilitate gallstone formation.^[12]^ Research indicates that IR leads to the production of cholesterol-supersaturated bile in high-risk Hispanic populations, which subsequently alters gallbladder function and contributes to the formation of gallstones.^[13]^ The formation of cholesterol gallstones was significantly predisposed in mice with isolated hepatic IR (LIRKO mice), which lack insulin receptors in the liver.^[14]^ Another in vivo experiment demonstrated that mice fed high-protein and high-quality diets developed sludge and gallstones more rapidly.^[15]^ The observation of increased IR symptoms following gallbladder removal during surgery has led to the hypothesis that the gallbladder may be involved in the regulation of insulin sensitivity.^[16]^ A new study has shown that the gallbladder acts as a regulator of bile acids kinetics and as a hormone-secreting organ, with metabolic actions at the systemic level.^[17]^ Another study conducted on 19,503 non-diabetic and non-obese Korean males revealed that IR was a significant predictor of gallstone disease and it was observed that a mutual association between GSD and IR.^[10]^ A recent study in US adults indicates a positive correlation between the METS-IR index and the prevalence of gallbladder stones.^[18]^ Additionally, a study has discovered a strong correlation between metabolic syndrome and the occurrence of cholelithiasis.^[19]^

HOMA-IR is a well-established and validated marker for assessing IR that has found extensive application in clinical settings. Despite its widespread use, 1 significant limitation arises from the fact that measuring insulin levels is not typically part of the standard evaluation for apparently healthy people. This lack of routine insulin measurement restricts the clinical utility of HOMA-IR in this specific population. In contrast, TyG index, as a new surrogate indicator of IR, is widely used to predict the risk and severity of metabolic syndromes, cardiovascular diseases, and cerebrovascular diseases.^[20–22]^ The TyG index calculation does not necessitate insulin quantification, rendering it suitable for all individuals. This index, which is derived from fasting triglyceride and glucose levels, is considered both reliable and straightforward for indicating IR. Recent research has indicated that the predictive capacity of the TyG index for identifying IR may surpass that of HOMA-IR, making it a potentially more effective tool in clinical practice, particularly in cases where insulin testing is not feasible.^[23–25]^ Reproducible IR surrogates such as TyG-BMI, TyG-WC, and TG/HDL-C are affordable markers that can be easily obtained in primary care and routine clinical practice, may providing a more holistic evaluation of metabolic risk related to disease.^[26]^ Given the association between IR and gallstones, as well as the recognition of TyG, TyG-WC, TyG-BMI, and TG/HDL-c as reliable indicators of IR, it is reasonable to speculate a positive relationship between these 4 IR surrogates and gallstones.

As far as we are aware, this is the initial research investigating the correlation between the IR surrogates and gallstones, provides preliminary confirmation that TyG, TyG-WC, and TyG-BMI are independent risk factors for the prevalence of GSD. The exact reasons for the differing between the 4 insulin surrogates are not fully understood. Additionally, no significant association was found between TC and low-density lipoprotein cholesterol levels with GSD risk. This finding aligns with the recent meta-analysis conducted by Zhang.^[27]^ Despite this, the exact mechanisms behind this phenomenon are not yet fully understood. One potential explanation is that increased levels of HDL cholesterol in the blood may support the production of liver bile acid,^[28]^ ultimately lowering the cholesterol saturation index,^[29]^ and improving the solubility of cholesterol in bile.^[30]^ Consistent with previous studies, we found that triglyceride levels were significantly elevated in GSD.^[31,32]^, while HDL cholesterol levels were low. Cavallini et al demonstrated that elevated serum triglycerides were linked to higher cholesterol saturation index^[33]^ and accelerated cholesterol crystal nucleation,^[28]^ both important processes in gallstone formation.

Subgroup analysis revealed significantly higher levels of TyG-WC and TyG-BMI in both genders with gallbladder stones, although not all were statistically significant. Previous studies have documented variations in IR between the sexes in humans and rodents. It has been observed that females tend to exhibit greater sensitivity to insulin compared to males.^[34]^ Wang et al^[35]^ have suggested a stronger link between visceral fat and serum triglycerides in women than in men. In females, there was a positive relationship between glucose-stimulated disposal risk and triglyceride levels, whereas this association was not observed in males.^[36]^ In addition, research has shown that women have a more pronounced connection with IR than men.^[37]^

A ROC analysis was conducted to assess the diagnostic value of TyG index, TyG-WC, TyG-BMI, and TG/HDL-C for gallstone disease. Gender differences in the ROC results showed that the 4 surrogate markers exhibited better predictive ability for gallstone disease in women compared to men. Previous study results have also shown that the impact of TyG-BMI and TyG-WC on hypertension is significantly greater in women compared to men,^[38]^ which is similar to our findings. The analysis suggested that TyG-WC may be the most effective and reliable marker among the 4 surrogates for assessing the risk of gallstone disease. Variations in sex hormones, metabolic control, body fat composition, lipid metabolism, and IR between males and females may contribute to differences in susceptibility to GSD related to metabolic diversity.^[39]^ A study found that TyG-WC was more effective in predicting diabetes than TyG and TyG-BMI.^[40]^ A new study have confirmed that TyG-WC is a reliable indicator for the presence of nonalcoholic fatty liver in Korean adults.^[41]^ WC is a useful indicator of abdominal obesity and is closely associated with disturbances in glucose and lipid metabolism. Visceral adipose tissue releases proinflammatory cytokines and adipokines, leading to IR. Moreover, WC is strongly linked to increased mortality risk, independent of BMI adjustments. The results indicate that WC could be a better predictor of IR and metabolic issues compared to BMI.^[42–44]^ Age is a crucial factor in the prevalence of GSD.^[45]^ When IR surrogates are combined with age, the area under the ROC curve can reach 0.734 in females and 0.684 in males, enhancing the early diagnosis rate of gallstones.

This study has several limitations as it was a cross-sectional clinical study, and limited by the lack of information on family and parity history. This study design does not allow for establishing a causal relationship between the IR surrogates and GSD, therefore further prospective cohort studies are necessary to confirm this relationship. Our research revealed a moderate level of significance in relation to the AUC value. Although the AUC range of 0.6 to 0.7 is considered clinically beneficial,^[46–48]^ further research with a larger sample size is required to confirm the results and investigate the potential of the 4 IR surrogates in evaluating the progression and prognosis of GSD. In conclusion, our study provided a simple and valuable marker for predicting the GSD. We hypothesize that early treatment and management of IR may help reduce the occurrence of GSD.

## 5. Conclusions

Our study has presented evidence indicating that TyG-WC and TyG-BMI are increasingly recognized as an independent predictor of GSD, with higher levels of IR surrogates being strongly associated with the presence of GSD, especially in females.

## Author contributions

**Conceptualization:** Xueqing Liu.

**Data curation:** Zixuan Hu, Zuoyang Zhang.

**Formal analysis:** Zuoyang Zhang.

**Software:** Xiaohui Zhang.

**Writing – original draft:** Jia Liu, Dele Bo.

**Writing – review & editing:** Jia Liu.
